# Supplementary material for: Biogeographic Patterns of Leaf Element Stoichiometry of Stellera chamaejasme L. in Degraded Grasslands on Inner Mongolia Plateau and Qinghai-Tibetan Plateau
Source: Plants (Basel). 2022 Jul 26;11(15):1943. doi: 10.3390/plants11151943 (PMC9370359; doi:10.3390/plants11151943)

Table S1 Leaf element contents and stoichiometry of *S. chamaejasme* in different sample sites in this study (Mean ± SD, n = 3)

Sample Sites		Leaf								
		C（g • kg^{-1}）	N（g • kg^{-1}）	P（g • kg^{-1}）	K（g • kg^{-1}）	C:N	C:P	N:P	N:K	K:P
Inner Mongolia Plateau	GY	496.84±19.91	19.68±0.79	2.11±0.08	5.31±0.16	25.25±0.00	235.13±0.03	9.31±0.00	3.71±0.20	2.51±0.13
	DL	512.12±28.08	22.86±1.41	2.78±0.15	5.64±0.47	22.50±2.62	184.86±20.44	8.22±0.34	4.08±0.58	2.04±0.27
	TPSQ	494.48±21.19	21.09±0.37	2.43±0.10	5.70±0.73	23.46±0.74	268.51±22.23	10.49±1.07	3.75±0.56	2.35±0.37
	SU	536.20±4.56	19.13±1.19	1.57±0.15	4.15±0.34	28.10±1.68	342.78±31.91	12.18±0.43	4.65±0.61	2.66±0.41
	SGDL	530.58±3.86	22.68±1.53	2.30±0.23	5.86±0.27	23.47±1.75	232.17±23.85	9.92±1.19	3.88±0.43	2.57±0.36
	XWQ	486.49±3.51	20.52±2.33	1.40±0.04	4.72±0.08	23.94±3.05	346.97±6.95	14.66±2.00	4.35±0.51	3.37±0.09
	BYXL	451.33±3.12	15.29±0.50	1.42±0.13	5.40±0.12	29.52±0.97	318.26±33.53	10.78±0.80	2.83±0.09	3.81±0.36
	BYH	505.43±2.84	19.72±0.62	1.89±0.15	6.10±0.41	25.64±0.74	268.51±22.23	10.49±1.07	3.25±0.26	3.23±0.37
	HTXL	485.75±2.96	17.39±0.80	1.79±0.11	5.86±0.36	27.93±1.11	271.93±15.67	9.74±0.29	2.97±0.11	3.28±0.03
	FYD	479.72±9.50	21.33±4.82	2.76±0.62	6.39	22.49±7.83	173.89±60.50	7.73	3.34	2.32
Qinghai- Tibet Plateau	YD	528.45±5.46	23.48±1.64	1.74±0.14	6.64±0.41	22.58±1.66	305.60±24.77	13.60±1.73	3.55±0.34	3.83±0.13
	TZ	498.50±7.70	20.35±1.79	2.13±0.08	5.57±0.70	24.60±1.73	234.73±8.45	9.57±0.66	3.67±0.15	2.62±0.29
	HC	497.96±1.32	16.60±6.76	3.19±0.16	9.08±0.92	33.17±11.85	156.60±8.04	5.29±2.44	1.88±0.94	2.85±0.18
	SD	507.82±5.16	21.82±1.93	1.77±0.06	7.78±0.38	23.38±1.90	287.56±9.63	12.35±1.00	2.80±0.20	4.40±0.08
	SN	492.53±5.21	20.22±1.20	2.30±0.12	8.06±0.46	24.40±1.27	214.05±11.12	8.77±0.28	2.52±0.26	3.50±0.27
	MT	502.91±2.39	20.70±4.79	1.40±0.07	8.61±0.49	25.25±6.18	359.69±17.93	14.75±3.23	2.42±0.63	6.15±0.27
	QL	524.76±2.28	19.14±0.91	1.99±0.14	7.94±0.06	27.46±1.41	264.21±17.64	9.65±0.95	2.41±0.12	4.00±0.30
	GC	483.47±16.03	18.90±2.50	2.25±0.06	6.38±0.58	25.82±4.18	215.19±10.71	8.47±1.27	2.99±0.47	2.84±0.22
	HY	490.98±7.10	17.86±1.24	1.98±0.08	6.66±0.45	27.57±1.99	234.54±9.06	8.49±0.81	2.68±0.02	3.38±0.37
	GH	504.30±5.96	18.24±1.37	2.15±0.11	6.02±0.20	27.75±11.99	234.54±9.06	8.49±0.81	3.03±0.25	2.80±0.05
	YTL	531.11±13.35	22.87±5.43	1.56±0.11	6.92±0.09	23.98±4.77	342.41±30.76	14.89±4.63	3.31±0.81	4.46±0.27
	HK	504.30±5.96	20.93±1.47	2.66±0.07	7.65±0.38	24.18±1.69	189.86±5.43	7.87±0.40	2.74±0.19	2.88±0.09
	TD	493.22±7.25	21.29±3.23	1.48±0.11	7.39±0.59	23.55±3.73	335.27±22.54	14.51±2.83	2.89±0.46	5.01±0.21
	ZK	469.81±17.14	19.93±2.57	2.52±0.27	7.80±0.49	23.79±2.58	188.10±26.72	7.99±1.68	2.57±0.46	3.10±0.14
	HN	529.02±5.35	21.15±2.01	2.95±0.24	8.10±0.38	25.15±2.15	180.40±16.35	7.20±0.81	2.61±0.12	2.76±0.25
	LQ	472.52±10.22	16.08±0.50	1.93±0.10	6.11±0.46	29.42±1.57	245.32±13.68	8.35±0.54	2.64±0.16	3.17±01.6
	REG	516.58±5.43	22.23±0.54	2.87±0.28	6.47±0.19	23.25±0.64	181.49±18.58	7.80±0.68	3.44±0.02	2.27±0.19
	TK	466.90±32.38	18.42±1.16	2.09±0.21	6.35±0.14	25.37±1.42	223.94±13.30	8.83±0.40	2.90±0.23	3.06±0.38
	HZ	465.01±16.53	18.67±2.69	3.01±0.22	5.98±0.51	25.36±4.74	154.99±10.23	6.26±1.34	3.15±0.65	1.99±0.08

Table S2 Soil physiochemical properties in different sample sites in this study (Mean ± SD, n = 3, n_{FYD} =1)

Sample Sites		Soil								
		C（$g\cdot kg^{-1}$）	N（$g\cdot kg^{-1}$）	P（$g\cdot kg^{-1}$）	K（$g\cdot kg^{-1}$）	C:N	C:P	N:P	N:K	K:P
Inner Mongolia Plateau	GY	74.86±1.22	5.56±0.30	0.75±0.02	14.37±1.40	12.61±0.89	95.60±3.80	7.58±0.24	0.37±0.03	20.72±1.52
	DL	60.57±4.68	4.46±0.31	0.63±0.01	22.84±0.30	13.60±0.91	97.01±9.47	7.14±0.65	0.20±0.02	36.53±0.40
	TPSQ	29.57±3.98	2.93±0.57	0.41±0.035	22.50±1.57	10.17±0.75	72.77±5.44	7.18±0.79	0.13±0.03	55.92±8.23
	SU	13.57±6.42	1.42±0.33	0.30±0.03	19.36±0.22	9.20±2.11	44.23±16.64	4.71±0.65	0.07±0.02	64.88±5.39
	SGDL	53.15±16.62	4.50±1.42	0.54±0.02	18.30±1.03	11.82±1.03	99.35±30.87	8.39±2.55	0.24±0.07	34.21±2.70
	XWQ	5.87±1.55	0.76±0.13	0.21±0.02	0.95±0.68	7.72±1.41	29.15±7.78	3.74±0.40	1.05±0.54	4.54±2.85
	BYXL	84.74±0.89	5.26±0.15	0.78±0.09	19.97±1.58	16.26±0.35	98.28±11.26	6.05±0.70	0.24±0.02	25.05±1.08
	BYH	13.80±0.85	1.5±0.08	0.34±0.03	18.69±0.71	9.20±0.21	40.61±2.52	4.41±0.18	0.08±0.00	55.02±3.14
	HTXL	27.29±0.659	2.72±0.091	0.35±0.02	19.30±0.54	9.96±0.10	81.14±3.76	8.15±0.38	0.15±0.01	54.31±2.35
	FYD	49.64	4.96	0.66	24.60	9.97	75.06	7.53	0.20	37.33
Qinghai- Tibet Plateau	YD	53.88±4.59	0.58±0.03	0.58 ±0.03	25.70± 2.38	11.31±1.90	93.56±7.92	8.49±2.10	0.19±0.05	44.53±1.90
	TZ	67.00±3.18	5.05±0.05	0.67 ±0.06	29.47 ±1.52	13.32±0.77	99.91±5.71	7.50±0.15	0.17±0.02	36.88±1.53
	HC	38.07±7.03	3.02±0.88	0.56 ±0.04	17.59 ±0.63	12.88±1.40	67.73±7.62	5.34±1.18	0.17±0.05	31.55±1.63
	SD	50.48±8.36	3.47±0.91	0.55 ±0.05	17.37 ±0.29	14.84±2.27	92.19±17.81	6.29±1.55	0.20±0.05	31.65±2.64
	SN	66.17±17.91	5.02±2.71	0.60 ±0.06	25.17 ±2.35	14.61±5.12	109.17±22.77	8.31±4.20	0.21±0.13	42.16±5.87
	MT	30.90±0.43	3.38±0.02	0.64 ±0.02	19.78 ±0.90	9.13±0.17	48.05±2.27	5.26±0.15	0.17±0.01	30.76±1.74
	QL	75.00±5.43	7.43±0.78	0.71 ±0.01	29.52 ±3.37	10.18±1.31	106.00±8.40	10.5±1.22	0.25±0.00	41.75±5.22
	GC	51.69±1.28	3.37±0.41	0.66 ±0.02	24.81 ±1.17	15.49±1.96	78.72±1.39	5.13±0.62	0.14±0.02	37.76±0.67
	HY	12.59±1.54	0.40±0.26	0.20 ±0.03	16.61 ±0.45	38.90±16.62	64.06±9.57	2.04±1.40	0.02±0.02	85.39±16.20
	GH	45.32±5.57	3.73±0.49	0.58 ±0.01	23.04 ±1.74	12.16±0.10	78.70±10.60	6.48±0.92	0.16±0.02	40.00±3.50
	YTL	9.82 ±2.51	0.24±0.13	0.40 ±0.05	20.74 ±0.04	45.21±11.73	24.87±8.20	0.61±0.39	0.01±0.01	52.13±6.41
	HK	74.42 ±8.91	5.60±0.96	0.57 ±0.04	26.12 ±5.75	13.38±1.17	130.24±16.69	9.77±1.42	0.22±0.07	45.48±8.34
	TD	35.87 ±6.64	3.34±0.78	0.58 ±0.04	14.86 ±1.14	10.82±0.77	62.01±15.29	5.79±1.76	0.22±0.05	25.52±2.67
	ZK	62.19 ±9.82	5.16±0.93	0.60 ±0.05	17.21 ±0.58	12.10±1.10	104.79±19.35	8.65±1.39	0.30±0.06	29.01±3.48
	HN	64.25 ±10.06	5.29±0.21	0.73 ±0.11	16.58 ±1.63	12.14±1.62	89.07±19.05	7.31±0.99	0.32±0.02	22.75±1.43
	LQ	53.95 ±7.05	4.58±0.36	0.72 ±0.03	26.40 ±0.25	11.76±0.60	75.40±10.66	6.40±0.61	0.17±0.02	36.88±1.53
	REG	38.43 ±7.74	3.57±1.13	0.82 ±0.12	30.55 ±0.99	11.09±1.60	48.08±13.94	4.50±1.73	0.12±0.03	37.89±6.11
	TK	44.14 ±14.63	3.48±0.92	0.72 ±0.04	22.75 ±0.88	12.54±0.83	61.89±21.63	4.88±1.36	0.15±0.05	31.85±2.10
	HZ	50.20 ±14.92	3.66±0.61	0.64 ±0.06	18.03 ±0.93	13.52±2.18	79.90±29.31	5.5±1.38	0.20±0.03	28.23±2.75

Sample Sites		Soil						
		AP (mg • kg^{-1})	AK (mg • kg^{-1})	AN (mg • kg^{-1})	NN (mg • kg^{-1})	WC	pH	Ec (μs • cm^{-1})
Inner Mongolia Plateau	GY	6.62±2.26	97±18.36	12.03±0.73	9.93±0.36	0.21±0.02	8.40±0.31	813±172.53
	DL	3.97±2.59	179.67±79.22	12.49±3.70	20.13±7.26	0.20±0.01	8.46±0.049	587.67±170.53
	TPSQ	4.62±1.75	225.67±86.59	29.13±3.70	5.66±1.57	0.07±0.01	7.52±0.32	83±20.95
	SU	2.50±1.13	86.67±37.07	9.37±0.77	6.37±1.55	0.05±0.00	7.68±0.047	87±45.13
	SGDL	6.93±1.23	84.33±40.61	14.13±2.83	12.81±3.22	0.19±0.03	7.94±0.09	714.67±219.54
	XWQ	2.36±0.08	109.67±15.26	12.33±1.51	4.32±0.28	0.04±0.01	8.03±0.32	48.33±11.93
	BYXL	5.30±1.72	279.67±260.93	34.33±13.223	83.35±35.44	0.14	7.64±0.29	874.5±30.41
	BYH	2.54±1.42	155.67±24.01	21.04±1.31	3.13±0.90	0.05±0.00	6.923±0.06	40±6.56
	HTXL	2.81±0.24	240±36.7	28.73±3.64	6.14±1.83	0.19	6.85±0.17	117.07±14.67
	FYD	4.85	286.00	20.28	11.65	0.20	6.86	118.60
Qinghai- Tibet Plateau	YD	6.75 ±1.38	89.33±49.41	42.43±2.25	10.42±3.12	0.26 ±0.04	7.81 ±0.22	195.00±36.76
	TZ	4.85 ±2.84	118.00±67.56	13.49±5.25	16.75±3.54	0.28±0.01	8.26 ±0.09	425.33±254.03
	HC	6.75±1.08	222.33±102.05	21.45±4.00	14.41±2.60	0.18±0.03	8.15±0.08	178.67±4.73
	SD	5.03 ±1.04	179.67±29.02	13.74±1.88	10.79±1.77	0.16 ±0.02	8.32±0.05	192.00±10.82
	SN	7.61±3.47	137.51±39.85	14.74±2.29	13.41±1.67	0.24 ±0.01	8.33 ±0.04	207.00 ±30.32
	MT	5.94 ±1.89	367.33±51.23	24.77±18.46	22.03±3.60	0.20±0.01	7.81 ±0.12	205.33±21.03
	QL	7.70±2.19	369.33±26.10	21.02±0.45	18.35±2.83	0.35 ±0.02	7.92 ±0.11	243.00 ±30.05
	GC	6.62 ±1.51	407.33±16.50	16.75±5.52	14.95 ±0.60	0.18 ±0.01	8.22 ±0.18	191.67 ±7.77
	HY	3.81±1.41	97.89±9.77	16.04±1.62	5.10 ±1.45	0.03 ±0.01	8.81 ±0.15	76.33 ±3.21
	GH	5.39 ±0.95	188.33±82.48	14.64±2.93	15.83 ±0.96	0.16±0.00	8.19±0.19	168.00±22.27
	YTL	3.04±0.83	86.67±34.02	7.64±3.05	6.01±1.02	0.06±0.02	8.30 ±0.05	128.33±4.51
	HK	6.93±0.31	63.67±11.24	25.59±2.68	12.60 ±0.92	0.31 ±0.04	8.28 ±0.09	192.67 ±13.65
	TD	2.72 ±0.90	134.00±53.70	12.02±3.74	14.57±2.13	0.17 ±0.01	8.27 ±0.15	155.67 ±26.63
	ZK	7.88±0.16	179.33±78.83	22.20±0.78	12.89 ±4.41	0.24 ±0.00	7.87 ±0.01	226.67 ±85.91
	HN	10.42±3.43	207.67±36.14	16.34±1.97	18.19±6.42	0.23±0.03	7.90 ±0.11	213.33±13.20
	LQ	3.90±0.76	205.33±38.28	20.83±5.02	13.22±4.73	0.26±0.02	7.98±0.05	184.00±10.44
	REG	4.76 ±0.55	196.67±98.90	12.34±4.60	11.35±1.88	0.23 ±0.01	7.89 ±0.11	202.00±21.63
	TK	4.71±1.47	42.33±23.16	18.95±0.96	5.96 ±0.47	0.23±0.02	6.82 ±0.37	56.67±2.31
	HZ	6.03 ±1.68	64.33±53.14	27.01±6.09	9.18 ±4.73	0.21 ±0.01	7.75±0.06	243.67±37.07

Table S3 Relationship between *S. chamaejasme* leaf stoichiometry and soil physicochemical properties in Northern China (n = 29 sites, Pearson correlations). Significant correlation is indicated by an * (P < 0.05).

	Leaf C	Leaf N	Leaf P	Leaf K	Leaf C:N	Leaf C:P	Leaf N:P	Leaf N:K	Leaf K:P
**Soil C**	-18.27%	-7.46%	32.91%	20.65%	1.23%	-41.00%*	-41.43%*	-27.93%	-17.84%
**Soil N**	-6.84%	1.42%	33.63%	26.88%	-4.55%	-39.55%*	-38.01%*	-27.44%	-13.48%
**Soil P**	-18.04%	-3.07%	36.04%	26.50%	-3.62%	-39.05%*	-36.92%*	-32.17%	-8.86%
**Soil K**	13.94%	5.49%	27.48%	15.60%	1.58%	-33.14%	-34.12%	-19.02%	-15.60%
**Soil C:N**	10.00%	4.64%	-13.27%	8.04%	1.07%	14.15%	14.61%	-11.04%	16.98%
**Soil C:P**	-13.02%	-5.35%	32.08%	19.49%	0.80%	-42.41%*	-42.71%*	-24.97%	-21.84%
**Soil N:P**	0.23%	5.78%	27.21%	17.50%	-7.05%	-33.68%	-31.76%	-13.87%	-17.57%
**Soil N:K**	-17.30%	5.85	-8.91	-15.10	-16.77%	10.75%	16.44%	22.52%	-2.85%
**Soil K:P**	26.18%	-6.47	-8.05	-10.24	20.41%	3.87%	-4.88%	3.85%	-6.53%
**Soil available P**	10.11%	7.57%	51.31%*	55.79%*	-5.44%	-49.76%*	-45.28%*	-45.84%*	-10.60%
**Soil available K**	-12.79%	-23.16%	-0.63%	29.47%	22.58%	-0.24%	-8.26%	-38.57%*	24.78%
**Soil ammonium N**	-31.40%	-22.06%	-0.05%	11.19%	14.48%	-4.38%	-8.64%	-27.29%	9.09%
**Soil nitrate N**	-33.42%	-36.38%	-16.41%	-1.30%	32.67%	14.55%	1.12%	-20.79%	17.82%
**Soil WC**	-2.70%	4.86%	36.81%*	40.87%*	-7.27%	-40.77%*	-36.55%	-38.40%*	-5.66%
**Soil pH**	24.92%	16.07%	2.61%	20.08%	-6.21%	1.89%	6.34%	-6.51%	11.16%
**Soil Ec**	-6.50%	-2.94%	3.03%	-23.17%	3.88%	-6.12%	-9.18%	16.59%	-15.80%

Table S4 Summary of linear mixed effects model analyzing the effects of soil, MAT and MAP on variation of C, N, P, K concentrations in *S. chamaejasme* leaves, using Soil, MAP, MAT and their interaction as fixed effects. Region was taken as a random factor

Element contents	Parameter	*df*	SS	*F*-value	*P*-value	Deviation explained (%)
	**Soil**	(1,21)	0.00033314	0.7422	0.3987	17.49
	**MAP**	(1,21)	0.00024345	0.5424	0.4696	46.46
	**MAT**	(1,21)	0.00030390	0.6770	0.4199	13.89
**Leaf C**	**Inter(Soil:MAP)**	(1,21)	0.00054312	1.2100	0.2838	
	**Inter(Soil:MAT)**	(1,21)	0.00000022	0.0005	0.9827	22.16
	**Inter(MAP:MAT)**	(1,21)	0.00000504	0.0112	0.9166	
	**Inter(Soil:MAP:MAT)**	(1,21)	0.00000017	0.0004	0.9847	
	**Soil**	(1,21)	0.00024636	0.0855	0.7729	6.11
	**MAP**	(1,21)	0.00000483	0.0017	0.9677	15.28
	**MAT**	(1,21)	0.00144192	0.5001	0.4872	72.62
**Leaf N**	**Inter(Soil:MAP)**	(1,21)	0.00009122	0.0316	0.8605	
	**Inter(Soil:MAT)**	(1,21)	0.00002671	0.0093	0.9242	6.99
	**Inter(MAP:MAT)**	(1,21)	0.00131536	0.4562	0.5068	
	**Inter(Soil:MAP:MAT)**	(1,21)	0.00000554	0.0019	0.9654	
	**Soil**	(1,21)	0.038602	3.7802	0.06538 .	29.95
	**MAP**	(1,21)	0.018317	1.7938	0.19478	36.49
	**MAT**	(1,21)	0.017602	1.7237	0.20339	11.53
**Leaf P**	**Inter(Soil:MAP)**	(1,21)	0.010788	1.0565	0.31572	
	**Inter(Soil:MAT)**	(1,21)	0.000936	0.0917	0.76506	22.03
	**Inter(MAP:MAT)**	(1,21)	0.009449	0.9253	0.34704	
	**Inter(Soil:MAP:MAT)**	(1,21)	0.004360	0.4269	0.52059	
	**Soil**	(1,21)	0.0078280	2.9705	0.09992 .	11.98
	**MAP**	(1,21)	0.0211776	8.0362	0.01023 *	24.70
	**MAT**	(1,21)	0.0074617	2.8315	0.10757	51.46
**Leaf K**	**Inter(Soil:MAP)**	(1,21)	0.0046609	1.7687	0.19840	
	**Inter(Soil:MAT)**	(1,21)	0.0005863	0.2225	0.64211	11.85
	**Inter(MAP:MAT)**	(1,21)	0.0026013	0.9871	0.33182	
	**Inter(Soil:MAP:MAT)**	(1,21)	0.0009943	0.3773	0.54596	

Note: Firstly, the soil data (soil C, N, P, K, AP, AK, AN, NN, WC, pH, Ec) is processed by principal component analysis (PCA), then, the number of first axis is used as Soil parameter. Leaf C, N, P, K and climatic data (MAP and MATA) heterogeneity was log-transformed to linearized the data.

Table S5 Summary of the total effects on the leaf P and K of *S. chamaejasme* in Qinghai Tibet Plateau (QT) and Inner Mongolia Plateau (IM). Significant effect is indicated by an * ($P < 0.05$).

Leaf elements	Relationships	QT			IM		
		Direct	Indirect	Total	Direct	Indirect	Total
P	**Climate → Soil**	0.369	0	0.369	0.682*	0	0.682
	**Climate → Leaf P**	-0.456	0.192	-0.264	0.665	0.123	0.788
	**Soil → Leaf P**	0.519*	0	0.519	0.180	0	0.180
K	**Climate → Soil**	0.331	0	0.331	0.590	0	0.590
	**Climate → Leaf K**	-0.476*	-0.127	-0.604	0.248	0.181	0.428
	**Soil → Leaf K**	-0.385	0	-0.385	0.306	0	0.306

Table S6 The geographical and climatic information associated with the sample sites in this study

Sample Sites		Latitude (N)	Longitude（E）	Altitude (m)	Mean Annual Precipitation(mm)	Mean Annual temperature(℃)
Inner Mongolia Plateau	**GY**	41.75	115.68	1380	354.77	3.69
	**DL**	41.81	116.09	1440	364.97	3.71
	**TPSQ**	41.82	115.30	1330	346.20	3.62
	**SU**	42.35	116.05	1310	348.86	3.30
	**SGDL**	42.67	115.95	1320	330.78	3.24
	**XWQ**	44.52	117.39	1060	314.09	2.73
	**BYXL**	43.76	116.79	1270	315.13	3.57
	**BYH**	44.77	118.16	1130	338.60	3.07
	**HTXL**	43.42	116.13	1300	302.00	3.32
	**FYD**	41.34	115.84	1535	368.29	4.28
Qinghai- Tibet Plateau	**YD**	36.83	103.02	2770	397.87	2.97
	**TZ**	37.21	102.77	2910	409.07	1.29
	**HC**	38.04	101.56	2790	211.86	7.42
	**SD**	38.45	101.20	2590	184.31	7.24
	**SN**	38.80	99.68	2352	143.84	8.19
	**MT**	38.47	100.48	2450	172.12	7.49
	**QL**	38.09	100.34	2970	306.79	4.72
	**GC**	37.25	100.36	3230	309.93	4.54
	**HY**	37.16	100.54	3280	316.20	4.62
	**GH**	36.63	100.86	3380	324.25	4.63
	**YTL**	36.36	100.72	2920	326.19	4.36
	**HK**	36.18	100.56	2920	337.76	4.06
	**TD**	35.26	100.53	3080	437.63	2.67
	**ZK**	35.25	101.03	3480	448.18	3.00
	**HN**	34.56	101.90	3500	565.56	2.82
	**LQ**	34.63	102.44	3100	570.41	2.79
	**REG**	34.09	102.73	3180	581.52	2.70
	**TK**	33.35	102.50	3450	587.53	6.30
	**HZ**	34.84	103.01	3180	549.36	3.47

Table S7 Eigenvalues of the soil PCA

	Dim.1	Dim.2	Dim.3
**Soil C**	0.951833918	0.17592561	0.001408344
**Soil N**	0.948442603	-0.01691258	-0.117697961
**Soil P**	0.876422966	0.006796241	-0.070848591
**Soil K**	0.548248139	-0.361333737	-0.361518754
**Soil available P**	0.729714855	0.090356356	-0.214290518
**Soil available K**	0.348820193	-0.393338992	0.298437622
**Soil ammonium N**	0.3371304	-0.608668314	0.356402949
**Soil nitrate N**	0.542945037	0.111331856	0.753622997
**Soil WC**	0.843821733	-0.124203841	-0.415628277
**Soil pH**	0.068389758	0.794001662	-0.200996436
**Soil Ec**	0.568393102	0.540578085	0.453887649

Table S8 Loadings of the soil PCA

	Dim.1	Dim.2	Dim.3	Dim.4	Dim.5
**Soil C**	0.951833918	0.17592561	0.001408344	-0.124669386	-0.015735198
**Soil N**	0.948442603	-0.01691258	-0.117697961	-0.105047464	0.028368126
**Soil P**	0.876422966	0.006796241	-0.070848591	0.126340077	-0.190794411
**Soil K**	0.548248139	-0.361333737	-0.361518754	0.284590289	-0.420972227
**Soil available P**	0.729714855	0.090356356	-0.214290518	-0.116243049	0.537969641
**Soil available K**	0.348820193	-0.393338992	0.298437622	0.706365468	0.310669133
**Soil ammonium N**	0.3371304	-0.608668314	0.356402949	-0.425586385	0.204506267
**Soil nitrate N**	0.542945037	0.111331856	0.753622997	0.114755697	-0.098583496
**Soil WC**	0.843821733	-0.124203841	-0.415628277	-0.087398817	-0.021670303
**Soil pH**	0.068389758	0.794001662	-0.200996436	0.247217601	0.226739269
**Soil Ec**	0.568393102	0.540578085	0.453887649	-0.126697032	-0.224293954

Table S9 Site scores of the soil PCA

	Site	Region	PC1	PC2	PC3	PC4	PC5	PC6
**sit1**	HN		-0.72595	0.28799	0.334746	0.17289	1.63856	1.22785
**sit2**	YTL		1.26143	0.64481	0.252156	-0.62634	-0.68378	-0.54803
**sit3**	REG		-0.26035	-0.21965	0.76542	-1.05542	-1.25369	0.0943
**sit4**	QL		-1.33971	-0.6523	0.601135	-1.02064	0.55014	0.06746
**sit5**	MT		-0.14804	-0.69702	-0.633402	-1.03997	0.92273	0.11591
**sit6**	TD		0.46061	0.60948	0.013844	-0.24643	-0.37118	0.30562
**sit7**	HK		-0.70163	0.08509	0.945926	1.05185	0.08749	-1.24512
**sit8**	SD		0.183	0.56206	0.171867	-0.32891	0.34878	0.09953
**sit9**	GH		0.03596	0.22331	0.218977	-0.58539	-0.02117	-0.27394
**sit10**	HY	QT	1.41231	0.79821	-0.004262	-0.20048	0.66737	-1.70381
**sit11**	SN		-0.51039	0.46561	0.858663	-0.02378	0.29115	-0.37844
**sit12**	TK		0.06963	-0.90871	0.653799	1.06371	-1.21614	1.2438
**sit13**	TZ		-0.63138	0.55355	0.666559	-0.17008	-1.31339	-0.53645
**sit14**	LQ		-0.37971	-0.36189	0.420216	-0.41891	-0.76231	-0.36504
**sit15**	GC		-0.3208	-0.24933	-0.013302	-1.9032	0.88034	-0.08703
**sit16**	HZ		-0.08064	-0.11308	0.151405	1.31843	0.12866	-0.0341
**sit17**	HC		0.07394	0.03278	-0.075225	-0.19476	1.07398	-0.15652
**sit18**	YD		-0.51269	-0.98259	0.234836	1.70638	0.41028	-1.78272
**sit19**	ZK		-0.46228	0.00684	0.228315	0.60301	1.08615	0.48702
**sit20**	BYH		1.20031	-1.07116	-0.427407	0.29643	-0.50788	0.48178
**sit21**	TPSQ		0.55071	-1.02848	-0.35814	0.05731	0.39571	-0.69059
**sit22**	DL		-0.33942	1.03592	-0.207553	-0.5125	-0.92422	-0.20775
**sit23**	SU		1.29909	0.10922	0.017856	-0.13811	-0.79704	0.08999
**sit24**	SGDL	IM	-0.2596	1.06852	-0.150545	0.85998	-0.21535	0.71363
**sit25**	XWQ		1.78154	0.68628	-0.643096	0.4511	1.0474	0.87793
**sit26**	GY		-0.66308	1.7524	-0.05152	0.75093	-0.14649	1.19817
**sit27**	BYXL		-1.33424	0.2378	-3.479361	0.11844	-0.71105	-0.58571
**sit28**	HTXL		0.58751	-1.54605	-0.482117	0.41235	-0.16405	0.32864
**sit29**	FYD		-0.24612	-1.3296	-0.009788	-0.39788	-0.441	1.26361

Figure S1. Heat map of Pearson correlations among *S. chamaejasme* leaf P content, soil P and AP content, soil water content (SWC), mean annual precipitation (MAP), and altitude (ALT) in IM region

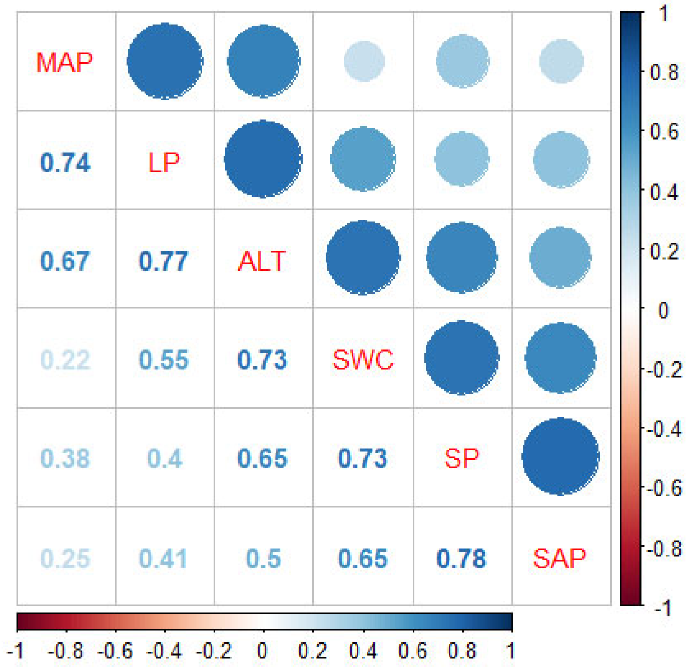

Figure S2. Biplot for the first two axes of soil physicochemical properties

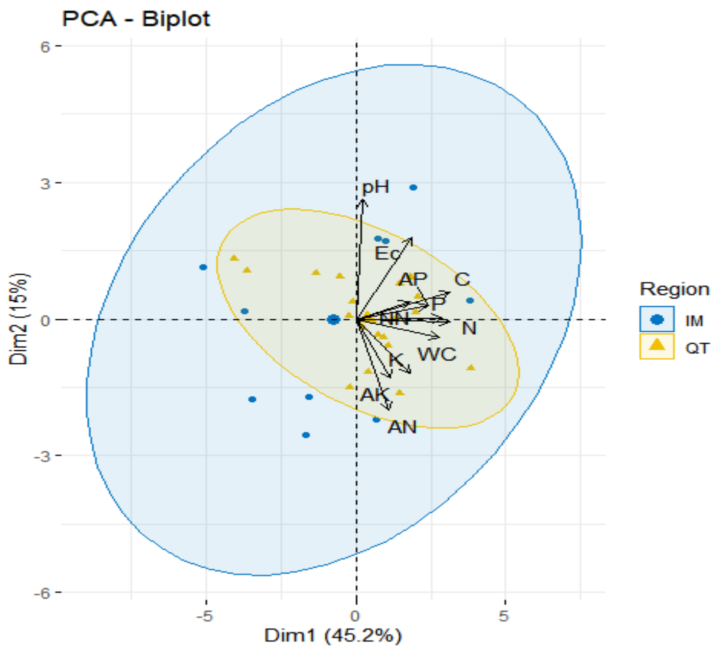

Supplement: Supplementary file 1 [file plants-11-01943-s001.zip › plants-1735226-supplementary.pdf]
